# Using a participation monitoring database to enhance recruitment in a rare cancer population

**DOI:** 10.1017/cts.2026.10703

**Published:** 2026-02-13

**Authors:** Michael A. O’Rorke, Brian M. Gryzlak, Tao Xu, Bradley D. McDowell, Rhonda R. DeCook, Nicholas J. Rudzianski, Kimberly C. Serrano, Abigayle M. Wehrheim, Udhayvir S. Grewal, Chandrikha Chandrasekharan, Joseph S. Dillon, Thorvardur R. Halfdanarson, Michael J. Schnell, Carrie L. Witter, T. Clark Gamblin, Syed Kazmi, Lindsay G. Cowell, Tobias Else, Heloisa P. Soares, Vineeth Sukrithan, Sravani Chandaka, Hanna K. Sanoff, Fiona C. He, David A. Geller, Robert A. Ramirez, Mei Liu, William Lancaster, Josh A. Mailman, Heather Moran, Maryann Wahmann, Elyse Gellerman, Elizabeth A. Chrischilles

**Affiliations:** 1 Epidemiology, The University of Iowa, USA; 2 The University of Iowa Holden Comprehensive Cancer Center, USA; 3 Internal Medicine, The University of Iowa Roy J and Lucille A Carver College of Medicine, USA; 4 Gastrointestinal Medical Oncology, The University of Texas MD Anderson Cancer Center, USA; 5 Medical Oncology, Mayo Clinic Minnesota, USA; 6 Mayo Clinic Minnesota, USA; 7 Medical College of Wisconsin, USA; 8 Internal Medicine, UT Southwestern Medical Center, USA; 9 O’Donnell School of Public Health, UT Southwestern Medical Center, USA; 10 Internal Medicine, Division of Metabolism, Endocrinology and Diabetes, University of Michigan, USA; 11 University of Utah Health Huntsman Cancer Institute, USA; 12 Division of Medical Oncology, Department of Medicine, The Ohio State University, USA; 13 The University of Kansas Medical Center, USA; 14 Division of Oncology, The University of North Carolina at Chapel Hill School of Medicine, USA; 15 Allina Health Cancer Institute, USA; 16 University of Pittsburgh, USA; 17 Department of Internal Medicine, Division of Hematology/Oncology, Vanderbilt University Medical Center, USA; 18 Health Outcomes and Biomedical Informatics, University of Florida, USA; 19 Surgery, Medical University of South Carolina, USA; 20 NorCal CarciNET Community, USA; 21 The Healing NET Foundation, USA; 22 Neuroendocrine Cancer Awareness Network, USA; 23 Neuroendocrine Tumor Research Foundation, USA

**Keywords:** Recruitment, neuroendocrine, representativeness, rare diseases, data collection

## Abstract

**Introduction:**

Recruitment for rare disease studies is challenging due to small eligible populations. Traditional clinical research management systems often lack tools to track recruitment contacts prior to enrollment. The NET-PRO study, focused on neuroendocrine tumors (NETs), implemented a participation monitoring system to enhance recruitment efficiency and representativeness.

**Methods:**

NET-PRO is a multicenter cohort study of 2538 adults diagnosed with gastroenteropancreatic (GEP) or lung NETs between January 2018 and September 2024. Recruitment occurred from January 2022 to February 2025 across 14 U.S. medical centers. Sites used flexible recruitment methods (email, mail, phone, in-clinic) and tracked contacts using REDCap-based tools. Participant characteristics were analyzed by enrollment mode (online or mail) and recruitment difficulty (number of contacts required prior to enrollment) using standardized mean differences, chi-square tests, and ANOVA.

**Results:**

Of 9279 contacted patients, 2675 consented (28.8%) and 2538 enrolled (27.4%). Most enrolled online (83.2%), while 16.8% enrolled by mail. Mail respondents were older, had lower education and income, and more comorbidities. Among those enrolled, recruitment difficulty was associated with older age, lower education and income, but not comorbidity. Over half of the most difficult-to-recruit participants enrolled online. Contact methods varied by attempt, with email dominating early contacts and phone/mail used more in later attempts.

**Conclusions:**

A participation monitoring tool supported flexible, multimodal recruitment and improved sample representativeness in a rare cancer study. Tracking recruitment contacts enabled adaptive strategies and may reduce bias in observational research by enabling better outreach to harder-to-reach populations.

## Introduction

Systematic tracking of participant contacts is essential for maintaining study validity, as incomplete contact documentation and loss to follow-up can introduce bias and undermine generalizability [1]. However, most clinical research management systems document study contacts only after enrollment begins [2,3]. This pattern likely reflects the absence of reporting requirements for pre-consent recruitment activity, as well as the highly variable nature of recruitment workflows. Indeed, identifying eligible patients often requires significant manual effort, with research teams frequently relying on ad hoc tools such as spreadsheets and email to track recruitment contacts longitudinally [4]. As a result, few tools support the systematic monitoring of outreach and contact attempts prior to enrollment, despite their importance for understanding recruitment efficiency, yield, and representativeness.

When implemented, recruitment monitoring tools can provide real-time insight into enrollment patterns, facilitate identification of hard-to-recruit subgroups, and enable adaptive strategies (e.g., re-contacting or switching recruitment modes) to enhance participation [5,6]. These capabilities are especially important in rare disease research, where recruitment inefficiencies driven by limited eligible populations, heterogeneous or fragmented care delivery, and variable site engagement constrain recruitment efficiency and yield [7,8]. This constrained and fragmented research landscape is also true in studies of neuroendocrine tumors (NETs), where available trials remain relatively few, are often small or early-phase, and are unevenly distributed geographically [9], underscoring the need for efficient, multi-site approaches to identifying, engaging, and enrolling patients across diverse care settings.

The Neuroendocrine Tumors – Patient Reported Outcomes (NET-PRO) study is a multicenter cohort designed to compare treatment approaches for gastroenteropancreatic (GEP) and lung NETs on patient outcomes [10]. To minimize nonparticipation bias, NET-PRO employed both online and postal recruitment and developed a centralized tool (Supplemental Material) to track and manage recruitment efforts across sites. This paper illustrates how recruitment monitoring data can be used to optimize enrollment processes and improve representativeness in rare disease research.

## Materials and methods

### Study population

NET-PRO is a cohort of 2,538 patients aged ≥18 years diagnosed with a GEP or lung NET between January 1, 2018, and September 30, 2024, identified across 14 medical centers from four PCORnet networks. Eligible patients had ≥1 clinical encounter at the recruiting site during this period; those with a prior NET diagnosis before 2018 were excluded. Additional exclusions included duplicate or ineligible consents and participants with missing or conflicting tumor site reports (Supplementary Figure 1).

Participating centers included Allina Health, Kansas University Medical Center, Mayo Clinic, Medical College of Wisconsin, Medical University of South Carolina, The Ohio State University, University of Florida, University of Iowa, University of Michigan, University of North Carolina, University of Pittsburgh Medical Center, University of Texas Southwestern, University of Utah, and Vanderbilt University Medical Center. The University of Iowa served as the coordinating center for the study and was the Institutional Review Board (IRB) of record for all sites (ID#: 202104599) except Vanderbilt University (ID#: 221155).

### Recruitment and enrollment

Enrollment began January 10, 2022, and concluded February 20, 2025. Enrollment was defined as completion of both informed consent and the baseline survey; individuals who consented but did not complete the baseline survey were not considered enrolled for analytic purposes. A validated computable phenotype was used to identify potentially eligible patients via electronic medical records (EMRs), with tailored versions for different strategies: a low-touch, high-specificity phenotype (for email/mail), a high-sensitivity version (for in-person/chart review), and a tumor registry-based approach leveraging structured oncology data. Patients identified via computable phenotypes or tumor registry queries were subsequently entered into the REDCap participation monitoring workflow, which linked case identification with outreach tracking and enrollment management.

Sites could use multiple outreach methods (email, phone, mail, in-clinic), depending on local resources, patient preferences, and regulatory requirements. Most patients were emailed a unique link to the study portal where informed consent was obtained and a baseline survey administered [11]. Sites also used mailed packets for patients without email, for those not responding to email or other approaches, or upon patient request.

A centralized participation monitoring project was developed using Research Electronic Data Capture (REDCap) tools hosted at The University of Iowa for tracking recruitment and enrollment events. REDCap is a secure, web-based software platform designed to support data capture for research studies [12,13]. Each site received a REDCap project template containing reports and a dashboard for case tracking that could be used to log patient contacts, track status (e.g., declines, withdrawals, deceased), and send email invitations. A subset of shared fields enabled sites to share recruitment data with the study coordinating center, while protecting patient personal health information (PHI). Similarly, individual sites could download a report from the study coordinating center containing status updates (e.g., patient enrolled or declined; patient reported as deceased, etc.) for import into their site project. This approach supported sites in near real-time to exclude cases from subsequent recruitment efforts and to monitor recruitment progress.

**Figure 1.**
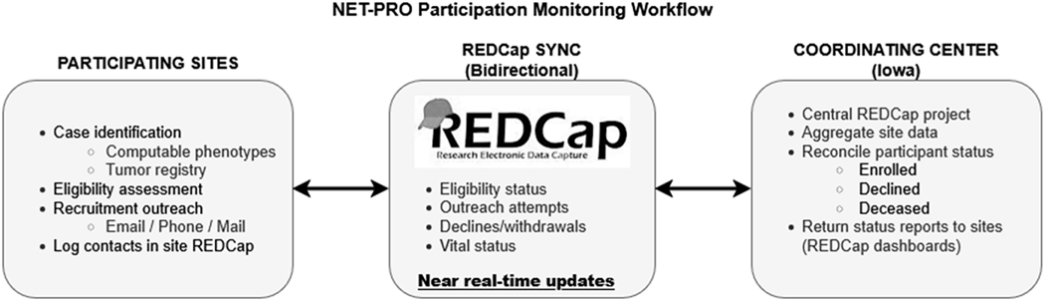
NET-PRO participation monitoring workflow. Participating sites identified potentially eligible patients using validated computable phenotypes, tumor registry queries, or manual chart review. Eligibility assessment and recruitment outreach (email, phone, mail, or in-clinic) were conducted locally and logged in site-level REDCap projects. A centralized REDCap participation monitoring project, hosted by the coordinating center, enabled bidirectional exchange of limited recruitment and status data, including eligibility confirmation, outreach attempts, enrollment, declines, and vital status. Near–real-time synchronization allowed sites to exclude patients who had already enrolled, declined participation, or were deceased, and supported adaptive recruitment strategies across sites. Enrollment was defined by completion of informed consent and baseline survey, either through the online study portal or via mailed materials.

Participating sites were responsible for local case identification, eligibility assessment, and recruitment outreach, including application of computable phenotypes, initiation of contact attempts, and logging of recruitment contacts, while the coordinating center hosted the centralized participation monitoring infrastructure, aggregated recruitment status data across sites, and returned updated enrollment status reports to support coordinated recruitment and prevent redundant outreach. The division of responsibilities and bidirectional flow of recruitment information are illustrated in Figure 1. All sites were encouraged to use the participation monitoring process, although implementation varied by local resources and regulatory constraints. The system was operationally validated through routine cross-site use, including reconciliation of enrollment status and exclusion of ineligible or previously enrolled patients from further outreach. Three sites (Iowa, MUSC, and UTSW) recorded all potentially eligible patients before screening; others logged only those they intended to contact. These three sites maintained comprehensive screening logs of all potentially eligible patients prior to outreach, reflecting local regulatory approvals and staffing capacity to document precontact eligibility; other sites recorded only patients they intended to contact due to institutional policies or resource constraints.

### Study variables and analysis

Surveys were completed online or by mail, with enrollment mode defined by the mode of informed consent and baseline survey completion (online study portal versus mailed paper materials), collecting a range of patient-reported data [10]. In-person consent was used infrequently in this study; “online” enrollment refers exclusively to consent and survey completion via the study portal. For this analysis, we assessed patterns in enrollment mode and recruitment difficulty (number of contact attempts before consent) across sociodemographic characteristics, tumor stage, and comorbidities. We also examined the mix of contact methods (e.g., email, phone, in-clinic, mail) across recruitment attempts (email, EMR message, phone call, in-clinic, letter, mailed study packet). Because participation-related factors may confound treatment-outcome associations, we explored differences in tumor stage and comorbidities by enrollment mode and recruitment difficulty, both potentially related to treatment choices and patient-reported outcomes.

We used standardized mean differences (SMDs) [14–17] to compare enrollment mode groups (with a SMD ≥ 0.2, 0.5, and 0.8 indicating small, medium, and large effect sizes, respectively) [18]. For recruitment difficulty (individuals who required multiple contact attempts before consenting to participate in the study), categorical variables were compared across difficulty groups using Pearson’s chi-square test or Fisher’s exact test, if any cell in the contingency table had fewer than five observations. Continuous variables were compared using one-way ANOVA.

## Results

### Response and completion rates

Of 9279 eligible patients contacted (range: 1–11 contacts overall; 1–9 among enrollees), 2675 consented (response rate: 28.8%), and 2538 enrolled (27.4%) (Figure 1, Table 1). Most enrolled online (*n* = 2,111; 83.2%) while 427 (16.8%) enrolled by mail. Among three sites that recorded all potentially eligible patients the proportion deemed eligible after medical record review ranged from 41.6% to 62.3%.

**Table 1.** Survey response and completion rates

	Overall
Number screened ^a^	11,286
Number who received at least one recruitment contact	9,279
Maximum number of recruitment contacts	11
Maximum number of recruitment contacts among enrolled patients	9
Median number of recruitment contacts	3
Median number of recruitment contacts among enrolled patients	2
Number who consented	2,675
Number who consented online	2,267
Number who consented by mail	408
Number who enrolled (completed baseline survey)	2,538
Baseline survey online	2,111
Baseline survey by mail	427
Eligible proportion ^a^	
Iowa	0.623
MUSC	0.539
UTSW	0.416
Response proportion ^b^	2675/9279 = 0.288
Enrolled proportion ^c^	2538/9279 = 0.274

^a^ Applies only to the three study sites that used the database to load all patients they intended to screen. Eligible proportion is the number contacted divided by the number screened at those sites.

^b^ Response proportion is the number who consented divided by the number contacted.

^c^ Enrolled proportion is the number who completed the baseline survey divided by the number contacted.

Iowa =University of Iowa, MUSC= Medical University of South Carolina, UTSW = University of Texas South Western.

### Participant characteristics by enrollment mode

There were substantial differences in sociodemographic and health status characteristics between those who enrolled online versus by mail. Mail enrollees were older (mean difference: 4.7 years; *p* < 0.001), had lower educational attainment and income (both *p* < 0.001), and reported more comorbidities (*p* < 0.001) than online enrollees (Table 2). Non-Hispanic Black participants were more likely to enroll by mail. There were no differences by sex or tumor site, though tumor stage was more often missing among mail respondents.

**Table 2.** Participant characteristics by enrollment mode

Characteristic	Overall	Enrolled online	Enrolled by mail	SMD*	*P*
*N* (row %)	2368	1982 (83.7)	386 (16.3)		
Sex, *N* (column %)				0.035	0.561
Female	1358 (57.3)	1142 (57.6)	216 (56.0)		
Male	994 (42.0)	827 (41.7)	167 (43.3)		
Unknown	16 (0.7)	13 (0.7)	3 (0.8)		
Race, *N* (column %)				0.298	<0.001
Hispanic (All Races)	68 (2.9)	58 (2.9)	10 (2.6)		
Non-Hispanic White	2089 (88.2)	1770 (89.3)	319 (82.6)		
Non-Hispanic Black	96 (4.1)	61 (3.1)	35 (9.1)		
Non-Hispanic American Indian/Alaska Native	10 (0.4)	5 (0.3)	5 (1.3)		
Non-Hispanic Asian/Pacific Islander	21 (0.9)	18 (0.9)	3 (0.8)		
Other	40 (1.7)	36 (1.8)	4 (1.0)		
Missing race	44 (1.9)	34 (1.7)	10 (2.6)		
Age, *N* (column %)				0.446	<0.001
18–39	270 (11.4)	248 (12.5)	22 (5.7)		
40–54	584 (24.7)	516 (26.0)	68 (17.6)		
55–64	650 (27.4)	552 (27.9)	98 (25.4)		
65–74	628 (26.5)	498 (25.1)	130 (33.7)		
75+	211 (8.9)	157 (7.9)	54 (14.0)		
Unknown	25 (1.1)	11 (0.6)	14 (3.6)		
Mean age (standard deviation)	57.66 (14.05)	56.84 (14.07)	61.97 (13.15)	0.376	<0.001
Education, N (column %)				0.543	<0.001
High school diploma, GED, or less	402 (17.0)	275 (13.9)	127 (32.9)		
College 1 year to 3 years	716 (30.2)	591 (29.8)	125 (32.4)		
College degree 4 years or more	680 (28.7)	604 (30.5)	76 (19.7)		
Postgraduate (Masters/PhD)	563 (23.8)	508 (25.6)	55 (14.2)		
Unknown	7 (0.3)	4 (0.2)	3 (0.8)		
Rurality, *N* (column %)				0.098	0.074
Rural	572 (24.2)	465 (23.5)	107 (27.7)		
Non-rural	1796 (75.8)	1517 (76.5)	279 (72.3)		
Number of comorbid conditions, *N* (column %)				0.269	<0.001
0	179 (7.6)	157 (7.9)	22 (5.7)		
1–2	766 (32.3)	675 (34.1)	91 (23.6)		
3+	1392 (58.8)	1124 (56.7)	268 (69.4)		
Unknown	31 (1.3)	26 (1.3)	5 (1.3)		
Summary stage at diagnosis				0.280	<0.001
Localized	919 (38.8)	759 (38.3)	160 (41.5)		
Regional	271 (11.4)	235 (11.9)	36 (9.3)		
Distant	781 (33.0)	684 (34.5)	97 (25.1)		
Missing	397 (16.8)	304 (15.3)	93 (24.1)		
Tumor location, *N* (column %)					
GEP NETs	1974 (83.4)	1660 (83.8)	314 (81.3)	0.063	0.245
Stomach	134 (5.7)	105 (5.3)	29 (7.5)		
Small Intestine	658 (27.8)	569 (28.7)	89 (23.1)		
Colon/rectum	161 (6.8)	131 (6.6)	30 (7.8)		
Appendix	122 (5.2)	109 (5.5)	13 (3.4)		
Pancreas	552 (23.3)	477 (24.1)	75 (19.4)		
GEP-NET, unspecified GEP location	137 (5.8)	125 (6.3)	12 (3.1)		
Missing	210 (8.9)	144 (7.3)	66 (17.1)		
Lung NET	394 (16.6)	322 (16.2)	72 (18.7)	0.063	0.245
Household income				0.639	<0.001
Less than $50,000	388 (16.4)	279 (14.1)	109 (28.2)		
$50,000 to $74,999	364 (15.4)	284 (14.3)	80 (20.7)		
$75,000 to $99,999	330 (13.9)	280 (14.1)	50 (13.0)		
$100,000 to $199,999	632 (26.7)	585 (29.5)	47 (12.2)		
$200,000 or more	325 (13.7)	292 (14.7)	33 (8.5)		
Prefer not to say	304 (12.8)	255 (12.9)	49 (12.7)		
Missing	25 (1.1)	7 (0.4)	18 (4.7)		

* Standardized mean differences (SMDs) were computed as the difference in means divided by the pooled standard deviation for continuous variables. For categorical variables, SMDs were calculated based on differences in proportions across groups (9-12).

N = Number of participants, GED = General Educational Development (high school equivalency), PhD = Doctor of Philosophy, NET = Neuroendocrine tumor, GEP NETs / GEP-NET = Gastroenteropancreatic neuroendocrine tumors, SD = Standard deviation, *P* = P-value, + = Indicates “or more.”

### Participant characteristics by recruitment difficulty

Most participants enrolled after one (41.8%) or two to three contacts (35.2%), while 14.3% required four to five, and 8.7% needed six or more contacts (Table 3). Participants who were more difficult to enroll, as measured by a higher number of recruitment contacts prior to enrollment, tended to be older, have lower income and education, and had higher rates of missing tumor site and stage data (Table 3). There was no clear pattern by tumor stage or GEP-NET location. Recruitment difficulty and enrollment mode were related but not synonymous. While harder-to-reach participants, individuals requiring a higher number of contact attempts prior to consenting to participate (reflecting increased recruitment effort rather than lack of interest or disengagement), were more likely to enroll by mail (*p* = <0.001), the majority of harder-to-reach participants nonetheless completed enrollment online.

**Table 3.** Influence of recruitment difficulty on enrolled participant characteristics

	Number of contacts required to enroll				
Characteristics	1	2 or 3	4 or 5	6 or more	*P*
*N* (row %)	989 (41.8)	834 (35.2)	339 (14.3)	206 (8.7)	
Sex, *N* (column %)					0.983
Female	565 (57.1)	478 (57.3)	199 (58.7)	116 (56.3)	
Male	418 (42.3)	350 (42.0)	138 (40.7)	88 (42.7)	
Unknown	6 (0.6)	6 (0.7)	2 (0.6)	2 (1.0)	
Race, *N* (column %)					0.065
Hispanic (All Races)	23 (2.3)	35 (4.2)	5 (1.5)	5 (2.4)	
Non-Hispanic White	888 (89.8)	721 (86.5)	307 (90.6)	173 (84.0)	
Non-Hispanic Black	33 (3.3)	37 (4.4)	12 (3.5)	14 (6.8)	
Non-Hispanic American Indian/Alaska Native	3 (0.3)	3 (0.4)	2 (0.6)	2 (1.0)	
Non-Hispanic Asian/Pacific Islander	5 (0.5)	12 (1.4)	2 (0.6)	2 (1.0)	
Other	16 (1.6)	14 (1.7)	7 (2.1)	3 (1.5)	
Missing race	21 (2.1)	12 (1.4)	4 (1.2)	7 (3.4)	
Age, *N* (column %)					0.117
18–39	127 (12.8)	91 (10.9)	37 (10.9)	15 (7.3)	
40–54	236 (23.9)	216 (25.9)	80 (23.6)	52 (25.2)	
55–64	269 (27.2)	237 (28.4)	94 (27.7)	50 (24.3)	
65–74	272 (27.5)	210 (25.2)	84 (24.8)	62 (30.1)	
75+	80 (8.1)	71 (8.5)	38 (11.2)	22 (10.7)	
Unknown	5 (0.5)	9 (1.1)	6 (1.8)	5 (2.4)	
Mean age (standard deviation)	57.33 (14.08)	57.30 (13.98)	58.62 (14.18)	59.10 (13.88)	0.188
Education, N (column %)					<0.001
High school diploma, GED, or less	123 (12.4)	139 (16.7)	78 (23.0)	62 (30.1)	
College 1 year to 3 years	298 (30.1)	228 (27.3)	117 (34.5)	73 (35.4)	
College degree 4 years or more	302 (30.5)	258 (30.9)	82 (24.2)	38 (18.4)	
Postgraduate (Masters/PhD)	264 (26.7)	208 (24.9)	60 (17.7)	31 (15.0)	
Unknown	2 (0.2)	1 (0.1)	2 (0.6)	2 (1.0)	
Rurality, *N* (column %)					0.202
Rural	232 (23.5)	201 (24.1)	77 (22.7)	62 (30.1)	
Non-rural	757 (76.5)	633 (75.9)	262 (77.3)	144 (69.9)	
Number of comorbid conditions, *N* (column %)					0.189
0	85 (8.6)	56 (6.7)	24 (7.1)	14 (6.8)	
1–2	323 (32.7)	289 (34.7)	97 (28.6)	57 (27.7)	
3+	568 (57.4)	475 (57.0)	216 (63.7)	133 (64.6)	
Unknown					
Summary stage at diagnosis					0.055
Localized	393 (39.7)	308 (36.9)	141 (41.6)	77 (37.4)	
Regional	114 (11.5)	92 (11.0)	38 (11.2)	27 (13.1)	
Distant	341 (34.5)	289 (34.7)	92 (27.1)	59 (28.6)	
Missing	141 (14.3)	145 (17.4)	68 (20.1)	43 (20.9)	
Tumor type					0.033
Lung NETs	163 (16.5)	123 (14.7)	74 (21.8)	34 (16.5)	
GEP NETs	826 (83.5)	711 (85.3)	265 (78.2)	172 (83.5)	
GEP-NET location, *N* (column %)					0.011
Stomach	48 (4.9)	49 (5.9)	15 (4.4)	22 (10.7)	
Small intestine	278 (28.1)	240 (28.8)	90 (26.5)	50 (24.3)	
Colon/rectum	61 (6.2)	59 (7.1)	26 (7.7)	15 (7.3)	
Appendix	59 (6.0)	41 (4.9)	13 (3.8)	9 (4.4)	
Pancreas	249 (25.2)	191 (22.9)	70 (20.6)	42 (20.4)	
GEP-NET, unspecified GEP location	62 (6.3)	52 (6.2)	14 (4.1)	9 (4.4)	
Missing	69 (7.0)	79 (9.5)	37 (10.9)	25 (12.1)	
Household income					<0.001
Less than $50,000	150 (15.2)	108 (12.9)	79 (23.3)	51 (24.8)	
$50,000 to $74,999	135 (13.7)	126 (15.1)	64 (18.9)	39 (18.9)	
$75,000 to $99,999	144 (14.6)	116 (13.9)	39 (11.5)	31 (15.0)	
$100,000 to $199,999	282 (28.5)	241 (28.9)	71 (20.9)	38 (18.4)	
$200,000 or more	149 (15.1)	123 (14.7)	33 (9.7)	20 (9.7)	
Prefer not to say	123 (12.4)	114 (13.7)	46 (13.6)	21 (10.2)	
Missing	6 (0.6)	6 (0.7)	7 (2.1)	6 (2.9)	
Enrollment mode					<0.001
Online	949 (96.0)	740 (88.7)	226 (66.7)	67 (32.5)	
Paper	40 (4.0)	94 (11.3)	113 (33.3)	139 (67.5)	

*Categorical variables were compared across groups using Pearson’s chi-square test or Fisher’s exact test, if any cell in the contingency table had fewer than five observations. Continuous variables were compared using one-way ANOVA.

N = Number of participants**,** GED = General Educational Development (high school equivalency), NET = Neuroendocrine tumor, GEP NETs / GEP-NET = Gastroenteropancreatic neuroendocrine tumors, SD = Standard deviation**,** P = P-value**,** ANOVA = Analysis of variance, + = Indicates “or more.”

### Recruitment mode by contact attempt

**Figure 2.**
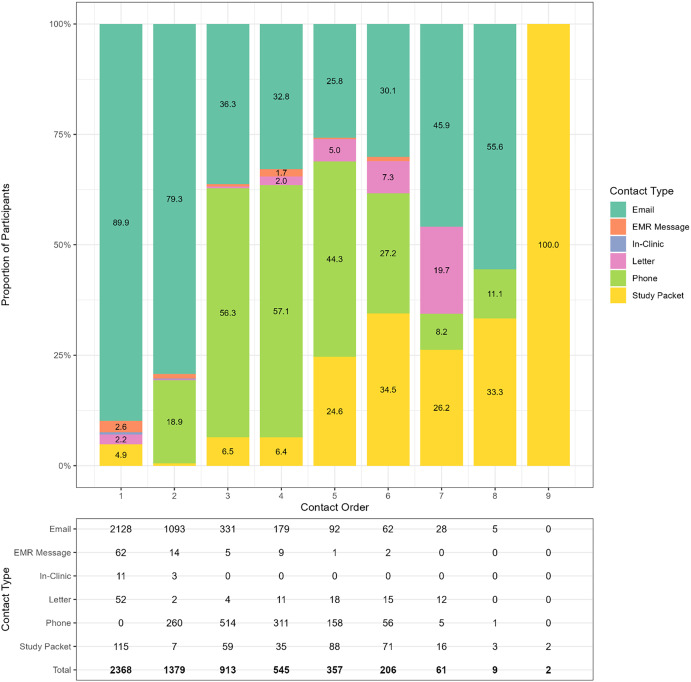
Stacked bar chart showing the distribution of contact types across successive contact attempts. The x-axis represents the contact order (1st attempt, 2nd attempt, etc.), while the y-axis indicates the proportion of each contact type (e.g., email, phone, letter, etc.) used at each attempt. The numbers in the data box indicate the number of participants per contact order by contact type.

Recruitment efforts evolved across contact attempts (Figure 2). While 89.9% of first contacts were via email, this dropped to 25.8% by the fifth attempt, then rose to 55.6% by the eighth. Mailed packets were typically initiated after the fourth attempt. Phone calls began as early as the second contact and accounted for over half of third and fourth contacts, highlighting their importance in engaging harder-to-reach participants and prompting consent among those not responsive to earlier email invitations.

## Discussion

In this multicenter PCORnet rare disease study, deploying multiple recruitment modes improved participation while supporting broader inclusion. The participation monitoring tool allowed sites to document recruitment contacts, determine when outreach should stop (e.g., after enrollment or decline), and adapt recruitment strategies over time.

Substantial sociodemographic differences were observed by enrollment mode and recruitment difficulty (Tables 2 and 3). Mail enrollees and harder-to-recruit participants were generally older, had lower educational attainment and household income, and were more likely to identify as non-Hispanic Black. These findings indicate that tracking recruitment effort and offering multiple recruitment pathways can facilitate inclusion of individuals who are often underrepresented in electronic-only recruitment strategies. Consistent with prior survey research, reliance on electronic enrollment alone may disproportionately exclude older adults and individuals with lower socioeconomic status [19,20]. While mixed-mode approaches may increase participation, they do not necessarily mitigate sociodemographic disparities [21]. These patterns are particularly salient in rare disease research, where small eligible populations, fragmented care pathways, and limited trial availability often necessitate sustained, multi-site recruitment efforts and adaptive outreach strategies [7,9,22].

In contrast, associations between clinical characteristics and enrollment mode or recruitment difficulty were modest. Mail enrollees reported more comorbidities, and participants requiring more contact attempts were more likely to have missing self-reported tumor stage data (Table 2). This is relevant for observational comparative effectiveness research, as factors such as comorbidity and tumor stage influence both treatment selection and outcomes (quality of life or tumor progression). The absence of strong or consistent differences in clinical characteristics between easier and harder-to-recruit participants may therefore be reassuring, suggesting limited selection bias with respect to overall health status (Table 3). The need for repeated contact among certain subgroups aligns with prior recruitment studies showing that varied and sustained outreach is often required to engage individuals who are not responsive to initial electronic invitations [19,21]. This pattern is clearly illustrated in Figure 2, which depicts the temporal evolution of outreach methods across successive contact attempts.

This study also demonstrates the feasibility of using EMR computer algorithms (also called computable phenotypes) to support recruitment in rare disease research. For the NET-PRO study, all participating sites used one or more computable phenotypes to identify potentially eligible patients, and three sites (Iowa, MUSC, and UTSW) recorded all patients screened for eligibility (Table 1). This allowed an estimation of the eligible proportions for a rare disease computable phenotype approach to recruitment. These proportions ranged from 42% to 62%, consistent with prior frameworks emphasizing that phenotype performance varies with clinical complexity, data availability, and algorithm design choices [23]. Thus, differences across sites likely reflect the specific phenotyping strategies employed, including high-touch (low sensitivity) and low-touch (high positive predictive value) EHR-based approaches and tumor registry or other site-specific data queries. Nevertheless, these findings support the feasibility of efficiently identifying eligible participants in a rare disease cohort.

Finally, this analysis also highlights the advantages of using REDCap for participation monitoring rather than developing bespoke software or licensing specialized commercial platforms [24]. REDCap is available at no cost to academic, nonprofit, and government organizations [13]. This widespread availability enabled the University of Iowa coordinating center to deploy a centralized monitoring project and share standardized site-level templates, facilitating harmonized tracking of recruitment and enrollment across sites.

Although assessment of representativeness was not a prespecified study aim, observed differences by enrollment mode and recruitment difficulty provide insight into how recruitment strategies may influence cohort composition. These findings are therefore interpreted cautiously as implications of recruitment processes rather than as formal assessments of population representativeness, particularly given the use of English-only recruitment materials and other pragmatic constraints. Additional limitations include the inability to estimate eligibility proportions at sites that did not track patients prior to eligibility confirmation, and reliance on self-reported tumor stage, though EHR-based stage abstraction is currently underway.

## Conclusion

In this multicenter study, a centralized participation monitoring tool supported substantial participation while enabling systematic documentation of recruitment effort and enrollment outcomes. By tracking recruitment contacts across sites and modes, this approach allowed adaptive outreach, reduced redundant contact, and facilitated empirical evaluation of how recruitment effort influenced cohort composition. In rare disease research, where eligible populations are limited and recruitment inefficiencies can substantially constrain study feasibility, this pragmatic, REDCap-based infrastructure offers a scalable solution readily transferable to other multi-site observational studies and pragmatic trials.

## Supporting information

10.1017/cts.2026.10703.sm001 O’Rorke et al. supplementary material O’Rorke et al. supplementary material

## Supplementary material

The supplementary material for this article can be found at https://doi.org/10.1017/cts.2026.10703.
