# Supplementary material for: Using a participation monitoring database to enhance recruitment in a rare cancer population
Source: J Clin Transl Sci. 2026 Feb 13;10(1):e40. doi: 10.1017/cts.2026.10703 (PMC12975625; doi:10.1017/cts.2026.10703)
Supplement: O’Rorke et al. supplementary material [file S2059866126107031sup001.pptx]

## Slide 1
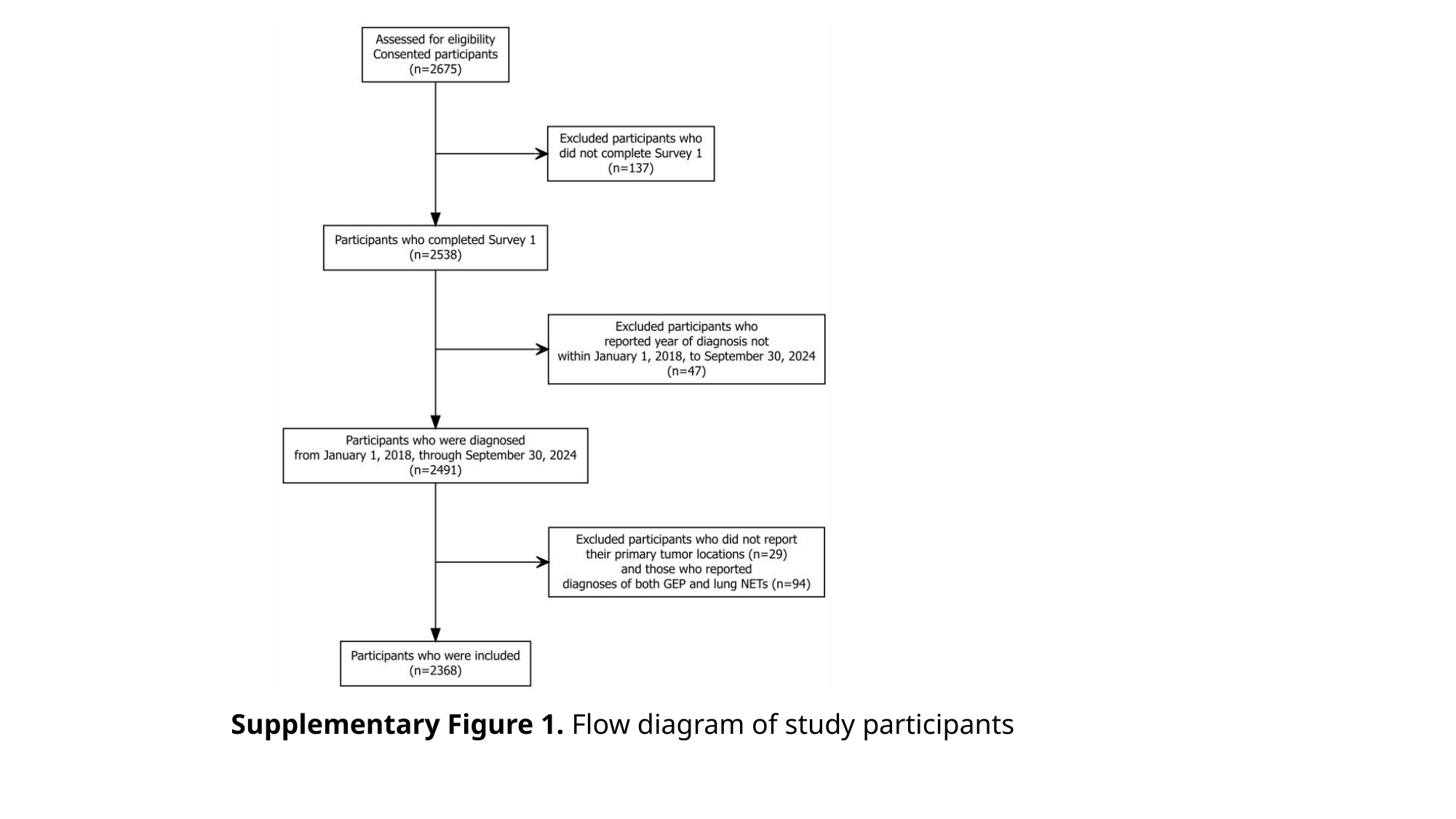

Supplementary Figure 1. Flow diagram of study participants

## Slide 2
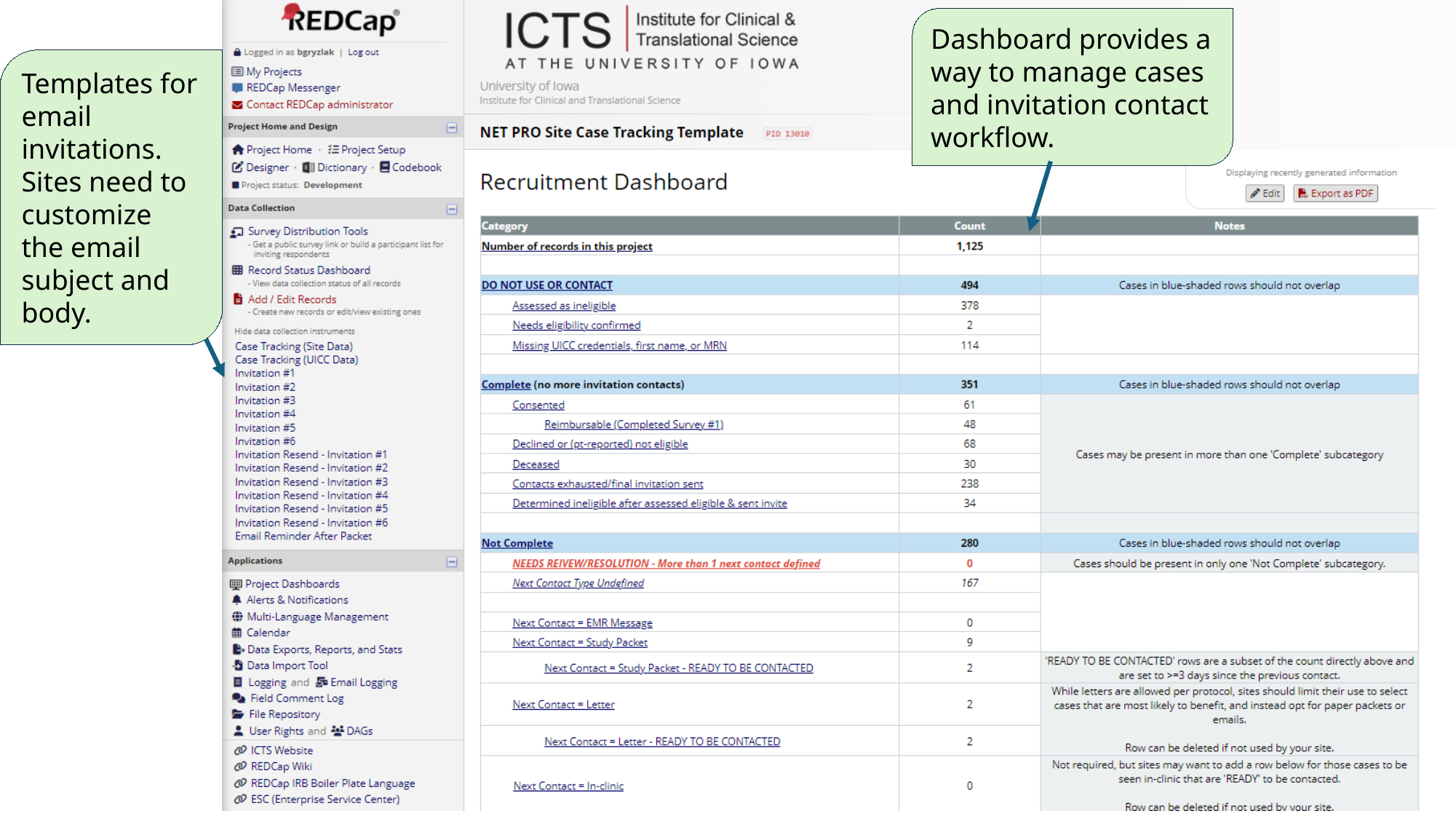

Dashboard provides a way to manage cases and invitation contact workflow.
Templates for email invitations.
Sites need to customize the email subject and body.

## Slide 3
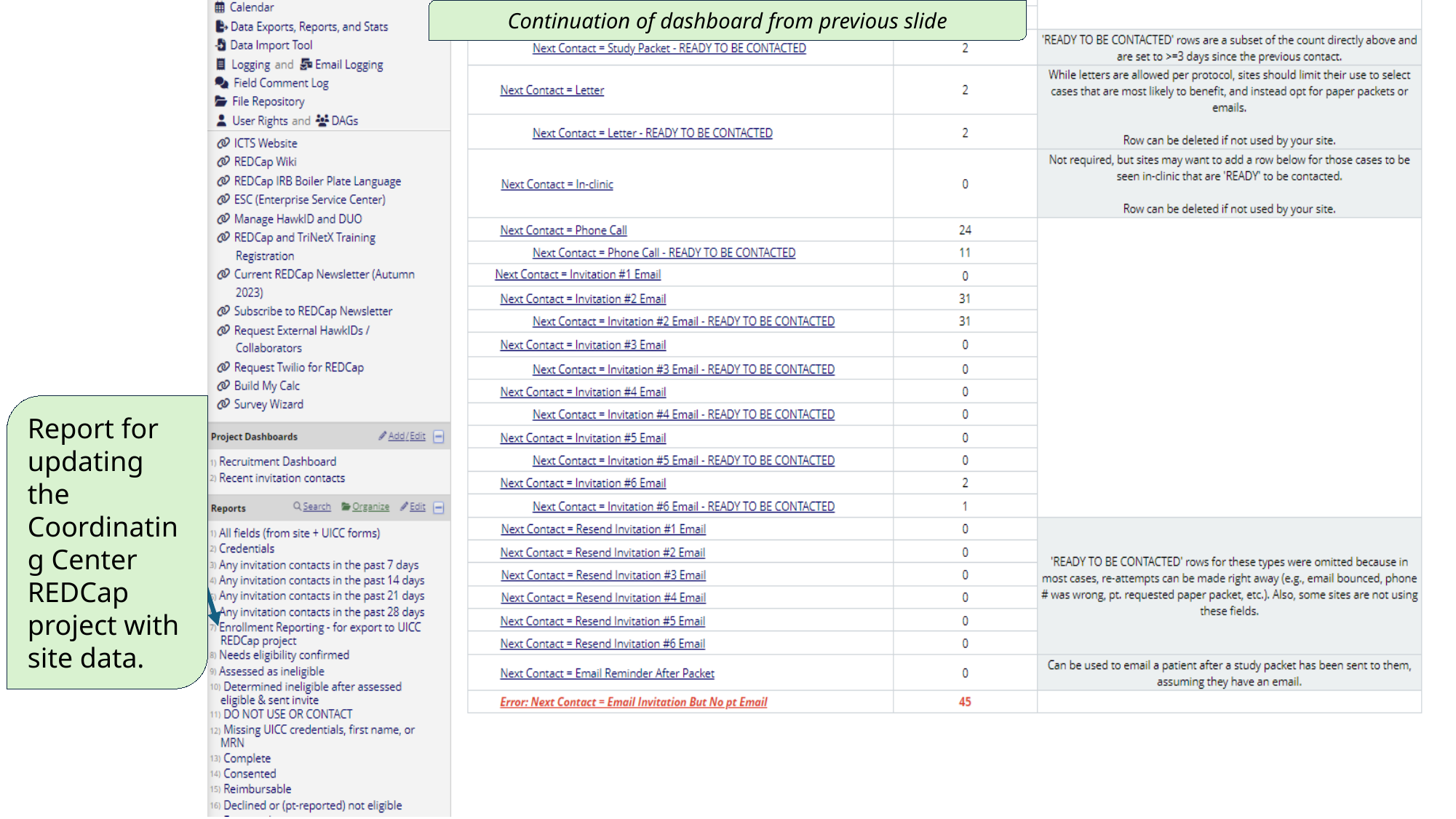

Continuation of dashboard from previous slide
Report for updating the Coordinating Center REDCap project with site data.

## Slide 4
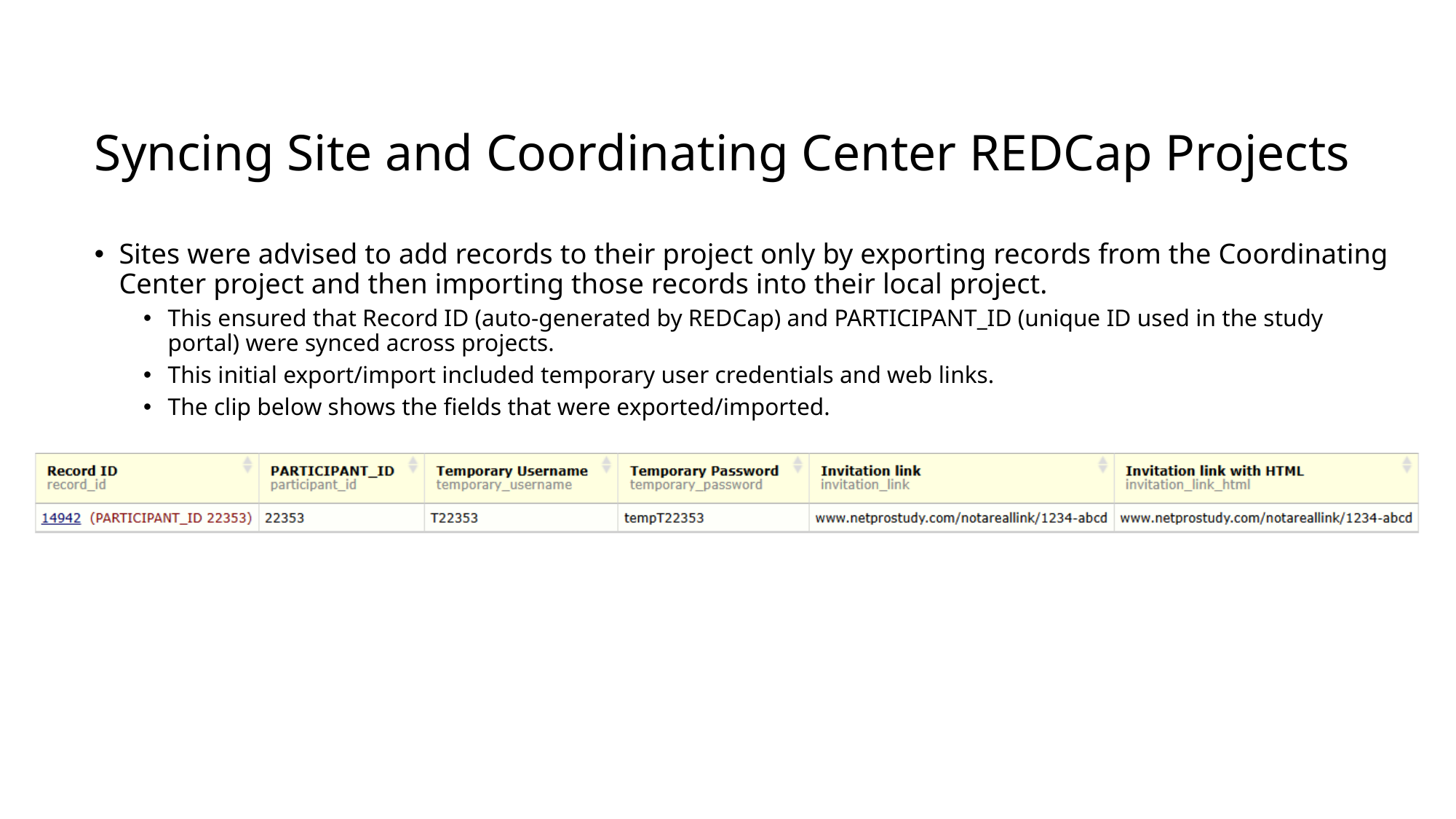

# Syncing Site and Coordinating Center REDCap Projects
Sites were advised to add records to their project only by exporting records from the Coordinating Center project and then importing those records into their local project.
This ensured that Record ID (auto-generated by REDCap) and PARTICIPANT_ID (unique ID used in the study portal) were synced across projects.
This initial export/import included temporary user credentials and web links.
The clip below shows the fields that were exported/imported.

## Slide 5
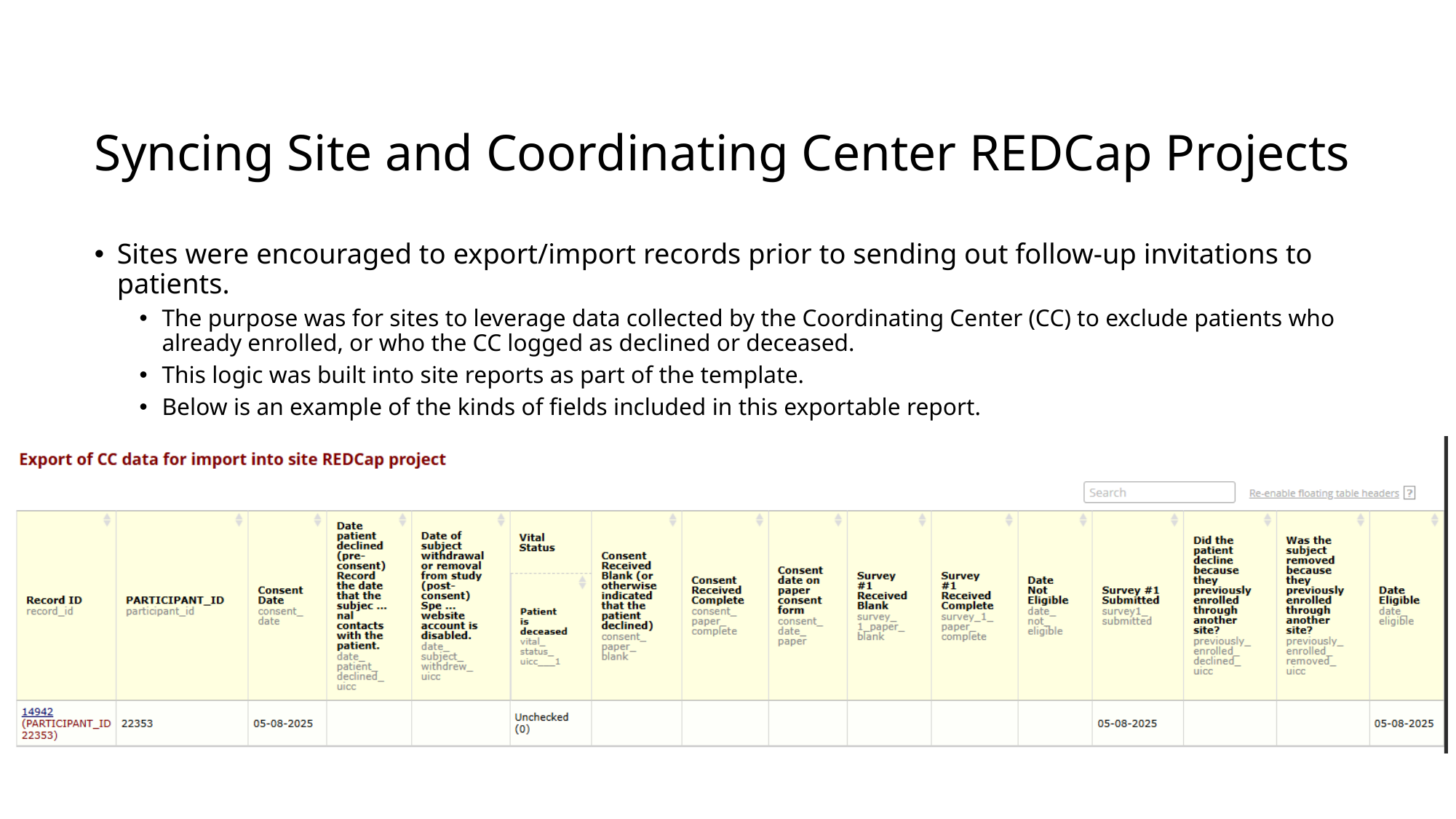

# Syncing Site and Coordinating Center REDCap Projects
Sites were encouraged to export/import records prior to sending out follow-up invitations to patients.
The purpose was for sites to leverage data collected by the Coordinating Center (CC) to exclude patients who already enrolled, or who the CC logged as declined or deceased.
This logic was built into site reports as part of the template.
Below is an example of the kinds of fields included in this exportable report.

## Slide 6
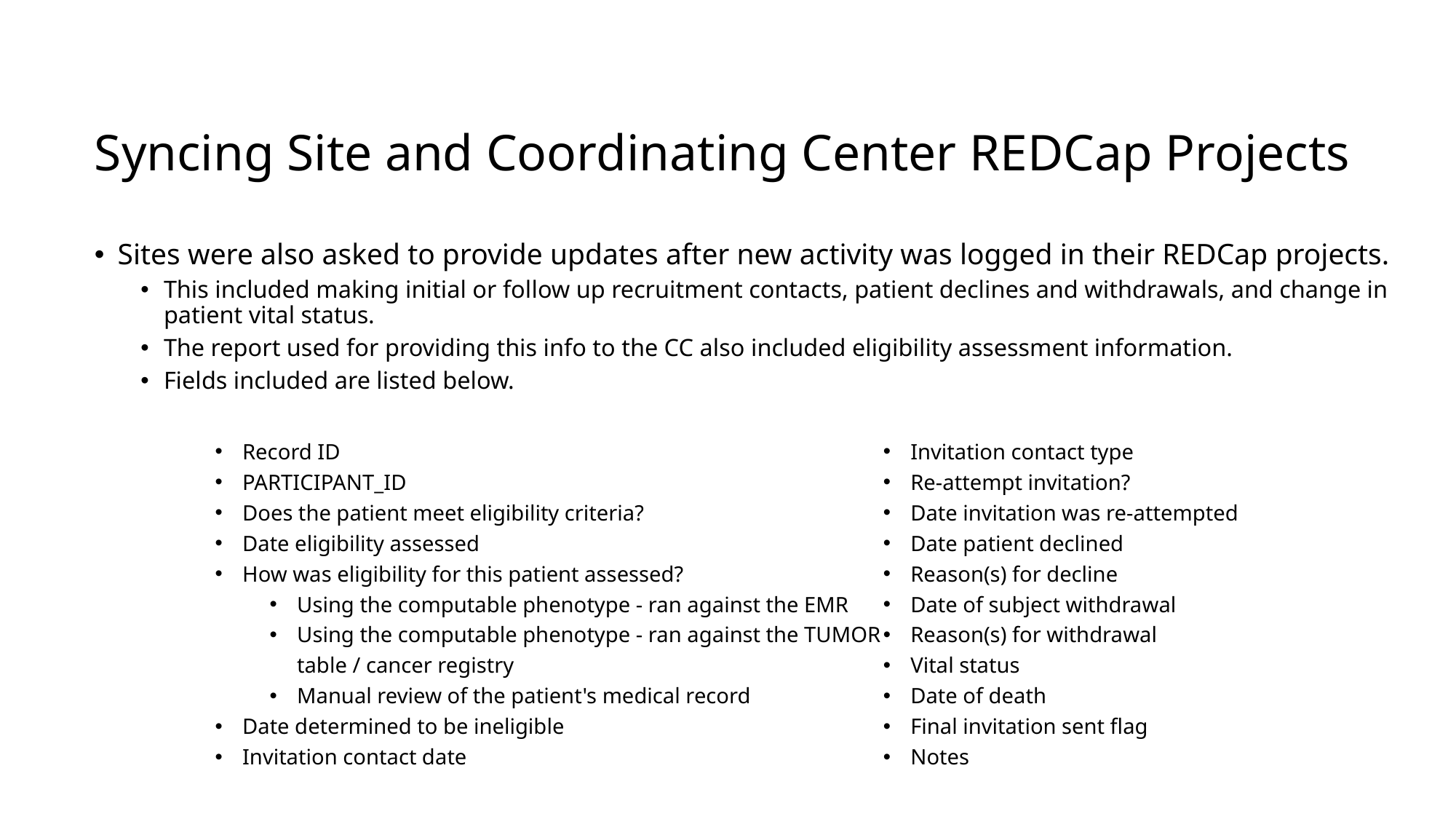

# Syncing Site and Coordinating Center REDCap Projects
Sites were also asked to provide updates after new activity was logged in their REDCap projects.
This included making initial or follow up recruitment contacts, patient declines and withdrawals, and change in patient vital status.
The report used for providing this info to the CC also included eligibility assessment information.
Fields included are listed below.
Record ID
PARTICIPANT_ID
Does the patient meet eligibility criteria?
Date eligibility assessed
How was eligibility for this patient assessed?
Using the computable phenotype - ran against the EMR
Using the computable phenotype - ran against the TUMOR table / cancer registry
Manual review of the patient's medical record
Date determined to be ineligible
Invitation contact date
Invitation contact type
Re-attempt invitation?
Date invitation was re-attempted
Date patient declined
Reason(s) for decline
Date of subject withdrawal
Reason(s) for withdrawal
Vital status
Date of death
Final invitation sent flag
Notes
